# Tobacco smoking and solid fuels for cooking and risk of liver cancer: a prospective cohort study of 0.5 million Chinese adults

**DOI:** 10.1002/ijc.33977

**Published:** 2022-03-03

**Authors:** Qiaorui Wen, Ka Hung Chan, Kexiang Shi, Jun Lv, Yu Guo, Pei Pei, Ling Yang, Yiping Chen, Huaidong Du, Simon Gilbert, Daniel Avery, Weijie Hu, Junshi Chen, Canqing Yu, Zhengming Chen, Liming Li

**Affiliations:** 1 Department of Epidemiology and Biostatistics, School of Public Health, Peking University Health Science Center, Beijing,100191, China; 2 Clinical Trial Service Unit & Epidemiological Studies Unit (CTSU), Nuffield Department of Population Health, University of Oxford, Oxford OX3 7LF, United Kingdom; 3 Oxford British Heart Foundation Centre of Research Excellence, University of Oxford, Oxford OX3 7LF, United Kingdom; 4 Peking University Center for Public Health and Epidemic Preparedness & Response, Beijing 100191, China; 5 Key Laboratory of Molecular Cardiovascular Sciences (Peking University), Ministry of Education, Beijing 100191, China; 6 Fuwai Hospital Chinese Academy of Medical Sciences, National Center for Cardiovascular Diseases, Beijing l00037, China; 7 Chinese Academy of Medical Sciences, Beijing 100730, China; 8 Medical Research Council Population Health Research Unit at the University of Oxford, Oxford OX3 7LF, United Kingdom; 9 Maiji Center for Disease Control and Prevention, Tianshui, Gansu 741020, China; 10 China National Center for Food Safety Risk Assessment, Beijing 100022, China

**Keywords:** Tobacco smoking, Solid fuel, Liver cancer, Prospective cohort study

## Abstract

Previous research found tobacco smoking and solid fuel use for cooking to increase the risk of chronic liver disease mortality, but previous cohort studies have not investigated their independent and joint associations with liver cancer incidence in contemporary China. The China Kadoorie Biobank (CKB) study recruited 0.5 million adults aged 30-79 years from 10 areas across China during 2004-2008. Participants reported detailed smoking and fuel use information at baseline. After an 11.1-year median follow-up via electronic record linkage, we recorded 2,997 liver cancer cases. Overall, 29.4% participants were current smokers. Among those who cooked at least once per month, 48.8% always used solid fuels (i.e. coal or wood) for cooking. Tobacco smoking and solid fuel use for cooking were independently associated with increased risks of liver cancer, with HRs (95% CIs) of 1.28 (1.15-1.42) and 1.25 (1.03-1.52), respectively. The more cigarettes consumed each day, the earlier the age of starting smoking, or the longer duration of solid fuels exposure, the higher the risk (P_trend_ <0.001, =0.001, =0.018, respectively). Compared with never smokers who had always used clean fuels (i.e. gas or electricity), ever-smokers who had always used solid fuels for cooking had a 67% (95% CIs: 1.29-2.17) higher risk. Among Chinese adults, tobacco smoking and solid fuel use for cooking were independently associated with higher risk of liver cancer incidence. Stronger association was observed with higher number of daily cigarette consumption, the earlier age of starting smoking and longer duration of solid fuel use.

## Introduction

Globally, liver cancer account for more than 810 thousand deaths each year [1], with 52% occurring in China alone, where liver cancer was the most commonly diagnosed cancer and the leading cause of cancer death in men under 60 [2]. Alcohol abuse, Hepatitis B and C virus (HBV, HCV) infections have been the major causes of liver cancer in China, estimated to account for 33%, 41%, and 8% deaths, respectively [1]. However, new HBV and HCV infections have declined dramatically in China due to successful HBV vaccination, mandatory HCV screening and other precautionary measures against blood-borne disease transmission [3, 4], so other modifiable risk factors are becoming increasingly relevant.

Notably, China has some of the world’s large population of active smokers (> 300 million) and solid fuel users (about 450 million) [5, 6]. Epidemiological studies have reported tobacco smoking as a risk factor for liver cancer [7], but they were mostly case-control studies or cohort studies conducted in Japan or other high-income countries where the stages of smoking epidemic differed substantially from that in China or other low- and middle-income countries. Previous cohort studies in China [8–14] were all carried out in the last century, lacking adjustment for key risk factors (e.g., HBV infection, alcohol consumption), and were based on specific population. The International Agency for Research on Cancer (IARC) has classified solid fuels smoke as a group 2A (probably carcinogenic to humans) and coal smoke as group 1 (carcinogenic to humans) for lung cancer [15]. In contrast, there is no evidence on the impact of solid fuel smoke on liver cancer.

Previously, we have discovered positive associations of smoking and long-term solid fuel use for cooking with risk of chronic liver diseases mortality among half a million adults in the China Kadoorie Biobank (CKB) study [16], but this was constrained by the lack of data on specific liver diseases, especially on hospitalization for liver cancer. In this study, we intended to explore the association of tobacco smoking and solid fuel cooking on the risks of liver cancer incidence with longer follow-up in the same population.

## Methods

### Study Population

Details of the CKB study have been described elsewhere [17, 18]. Briefly, 512,715 participants aged 30-79 years were recruited from 10 areas across China, including 5 urban regions (Harbin, Qingdao, Suzhou, Liuzhou, and Haikou) and 5 rural regions (Henan, Gansu, Sichuan, Zhejiang, and Hunan), during 2004-2008. Participants with missing body mass index (BMI, *n* =2), and those who reported potentially unreliable recall information (i.e. the difference between the total number of years lived in the 3 most recent residences and baseline age greater than 1, *n* =2,308), or those with a self-reported prior diagnosis of any cancer at baseline (*n* =2,578) were excluded, leaving 507,837 participants for the primary analysis. For the cooking-related analyses, we additionally excluded those who cooked irregularly (i.e. less than once per month) at baseline (*n* =129,845), reported using unspecified fuels at any recalled residences (*n* =3,463), switched from clean to solid fuels (*n* =304) or switched between clean and solid fuels back and forth in the last three residences before baseline survey (*n* =777), leaving 373,448 participants.

### Assessment of Exposure

Trained health workers administered a computer-assisted questionnaire (with built-in logic and error checks to avoid missing data and erroneous data entry) to collect detailed information about participants’ current and past smoking habits and cooking behavior in the 3 most recent residences where they had lived for at least a year.

All participants provided their current and past (only among those who reported not smoking at baseline) smoking frequency (not smoking, occasionally, on most days, daily or almost every day). Among ever smokers, additional information on the age (years) of starting smoking on most days, the types of tobacco and quantity of daily (factory filter cigarettes, factory non-filter cigarettes, hand-rolled cigarettes, cigars) or monthly (hand-rolled cigarettes, pipes or water pipes, liang per month, 1 liang equivalent to 50 g) consumption was collected. Equivalent number of daily cigarette consumption was calculated as (factory filter cigarettes + factory non-filter cigarettes + 2 × cigars + 5/3 × hand-rolled cigarettes + 5/3 × pipes or water pipes), by assuming a factory cigarette containing 1 gram of tobacco and a cigar containing 2 grams [19]. For ex-smokers, to avoid reverse causality bias, those who had stopped smoking because of illness were grouped with current smokers as per previous studies [20]. Participants were categorized according to smoking status (never smoker, ex-smoker, current smoker), number of cigarettes consumed per day (≤10, 11-20, >20), and the age of starting regular smoking (>25, 19-25, ≤18 years) among ever smokers.

For each eligible residence, we obtained information about participants’ duration of living (in years) and their corresponding cooking frequency (no cooking facility, never/rarely, monthly, weekly, daily). For participants who cooked at least once per month, we further asked their primary cooking fuel (gas, coal, wood, electricity, other) in each residence. “Gas” refers to natural gas, coal gas, and liquefied petroleum gas. The “other” category comprised all fuel types not specified above. We considered gas and electricity as clean fuels, whereas wood and coal as solid fuels. Although participants might have used multiple fuel types simultaneously, we only recorded the one used most frequently and for the longest duration in each residence. Based on the long-term cooking fuel use in the last three residences, participants were categorized into three groups (e.g., always clean fuels, solid to clean fuels, and always solid fuels). Long-term solid fuel users were also categorized according to the total duration of exposure (<15, 15-29, ≥30 years) and specific solid fuel types (always coal, always wood, mixed of coal and wood). For residences with cooking facilities, we further asked if all cooking stoves were equipped with a chimney or extractor (all stoves, not all stoves, none).

A composite exposure of smoking status (never- or ever- smoker) and long-term fuel use (always clean fuels, ever solid fuels) was derived to investigate the potential joint effects of both exposures, taking those who had never smoked and always used clean fuels for cooking as the reference group.

Within a few weeks of the baseline survey, about 3% (*n* =15,720) of participants were randomly selected to a quality control survey [18]. The kappa coefficients between the baseline and quality control survey were 0.94 for smoking and 0.61 for cooking fuel [21], indicating acceptable reliability.

### Assessment of Covariates

Through the same electronic questionnaire mentioned above, a variety of covariates were assessed at the baseline survey, including sociodemographic characteristics (e.g. age, sex, marital status, education, household income and occupation), lifestyle and dietary habits (alcohol drinking, physical activity, frequency of fresh fruit, preserved vegetables, red meat, fish and grains consumption), living environment (environmental tobacco smoke, storing pesticides at home, having a refrigerator at home, whether heating in winter and the fuel types used), medical history (diabetes, hepatic cirrhosis, cancer) and family medical history of cancer. The total daily physical activity level was calculated by multiplying the metabolic equivalent of tasks (MET) value for a particular type of physical activity by hours spent on that activity per day and summing the MET hours per day (MET-h/d) for all activities [22].

Body weight (kg) and standing height (m) were measured by uniformly trained staff using a standard protocol and calibrated instruments at baseline. BMI (kg/m^2^) was calculated by dividing weight in kilograms by the square of height in meters. For each participant, a 10-ml non-fasting blood sample (with time of last meal recorded) was collected and tested for hepatitis B surface antigen (HBsAg) (ACON Biotech).

### Follow-up and Outcome Definition

Through record linkage to death and disease registries and a national health insurance system, participants were followed up from baseline to the date of any liver cancer [International Classification of Diseases, 10^th^ Revision (ICD-10): C22] diagnosis, death (n=48,480), loss to follow-up (n=5,246), or 31 December 2017, whichever came first. Vital status and cause of death were ascertained through reviews of official residential records and death certificates submitted to the regional Center for Disease Control and Prevention. The national health insurance system was established in all study regions by 2009 and it documented detailed hospitalization information, including ICD-10 codes, dates of diagnosis, and procedures.

### Statistical Analysis

Linear regression and logistic regression were used to compare continuous and categorical baseline characteristics across baseline smoking status (never smoker, ex-smoker, current smoker) and long-term cooking fuel types (always clean fuels, solid to clean fuels, always solid fuels), respectively, adjusted for age, sex, and study areas as appropriate.

Cox proportional hazard regression was used to estimate adjusted hazard ratios (HRs) and 95% confidence intervals (95% CIs) for the independent associations of tobacco smoking (taking never smoker as the common reference group) and cooking fuel use (taking those who had always used clean fuels as the common reference group) with liver cancer incidence. Potential confounders were selected based on prior knowledge of liver cancer risk factors, and were adjusted for in a stepwise manner. In order to illustrate the impact of adjusting for different sets of key confounders, we presented three sets of models with increasing number of covariates. The basic models (model 1) were stratified by sex, baseline age groups (in 5-year intervals) and study region, and adjusted for education (no formal school, primary school, middle school, high school, college and above), household income (<10000, 10000–19999, ≥20000 CNY/year), occupation (manual, non-manual, not working) and marital status (married, widowed, separated or divorced, never married). Model 2 further adjusted for alcohol consumption [never/occasional, ex-regular, weekly but not daily, daily <15 grams per day (g/d), 15-29 g/d, 30-59 g/d, ≥60 g/d], environmental tobacco smoke [never lived with smoker, lived with smoker for <20 years, lived with smoker for ≥20 years and exposure <20 hour per week (h/w), lived with smoker for ≥20 years and exposure ≥20 h/w], months of storing pesticides at home (continuous, month), long-term heating fuel exposure (always used clean fuels, switched from solid to clean fuels, always used solid fuels, switched from clean to solid fuels, switched between clean and solid fuels back and forth, ever used unspecified fuels, not heating in winter), stoves with chimney/extractor (all stoves, not all stoves, none stoves), physical activity (continuous, MET-h/d), BMI (<18.5, 18.5-24.0, 24.0-27.9, ≥28.0 kg/m^2^ according to overweight/obesity definition of Chinese population [23]), having a refrigerator at home (never, 1-5, 6-10, 11-15, >15 years), consumption frequency of fresh fruit, preserved vegetables, red meat, fish and grains at baseline [never/rarely, monthly, 1-3 days per week (d/w), 4-6 d/w, daily], and mutually adjusted for long-term cooking fuel exposure (always used clean fuels, switched from solid to clean fuels, always used solid fuels, switched from clean to solid fuels, switched between clean and solid fuels back and forth, ever used unspecified fuels, cooked irregularly) and smoking habits (never/occasional, quit ≥5 years ago, quit <5 years ago, current <15 cigarettes per day, current 15-24 cigarettes per day, current ≥25 cigarettes per day). Model 3 further included HBsAg status [negative, positive, unclear, missing (n=8,149)], family history of cancer (yes or no), medical history of hepatic cirrhosis (yes or no) and diabetes (yes or no) at baseline. Only results from the “fully adjusted” model 3 are considered final and are quoted in the main text.

Stratified analyses by baseline characteristics, such as sex, region, BMI and physical activity, were performed to examine potential effect modifications. We conducted three sensitivity analyses separately: (1) excluding those who developed liver cancer during the first 2 years of follow-up; (2) excluding those with family history of cancer; and (3) adjusting for detailed alcohol consumption (11 groups: never, occasional, ex-regular, weekly but not daily, daily <15 g/d, 15-29 g/d, 30-44 g/d, 45-59 g/d, 60-74 g/d, 75-89 g/d, ≥90 g/d). All analyses were performed using Stata 15.0 (StataCorp, TX, USA). The significance level was set at 0.05.

## Results

Among 507,837 participants with a mean (SD) age of 51.5 (10.7) years, 59.0% were female, 55.8% resided in rural areas, 29.4% were current smokers, and 73.5% reported cooking regularly at baseline, of whom 48.8% had always used solid fuels for cooking. Current smokers and solid fuel users had less education and lower income, were less likely to be non-manual workers, but more likely to be alcohol drinkers, to use solid fuels for heating, and have poorer kitchen ventilation. Compared with those who had always used clean fuels for cooking, solid fuel users were more likely to be rural residents, current smokers, and exposed to environmental tobacco smoke.

During a median follow-up of 11.1 (interquartile range 10.2-12.1) years, we documented 2,997 incident liver cancer cases, of whom 24.6% were HBsAg positive compared to 2.9% in those who did not develop liver cancer. Compared with never smokers, current smokers had 28% (95% CIs: 1.15-1.42) higher risk of liver cancer. Besides, the association followed a dose-response trend with daily cigarette consumption and the starting age of regular smoking (P_trend_<0.001 and P_trend_ =0.001, respectively, Table 2). Compared to never smokers, the HRs of liver cancer incidence increased from 1.18 (1.03-1.36) among those who started smoking after 25 years old to 1.34 (1.17-1.53) among those who started at 18 or earlier.

Compared to long-term clean fuel users, long-term solid fuel users had a higher risk of liver cancer (HRs: 1.25, 95% CIs: 1.03-1.52). However, individuals who had switched from solid to clean fuels had no significant excess risk (1.10, 0.94-1.28). Among those who had always used solid fuels for cooking, wood users and mixed solid fuel users had higher risks, with HRs of 1.27 (1.04-1.56) and 1.34 (1.04-1.73), respectively, whereas long-term coal users had no significant excess risk (1.14, 0.88-1.47). A dose-response trend was observed between the duration of solid fuels exposure and liver cancer risks (P_trend_ =0.018, Table 2).

**Figure 1:**
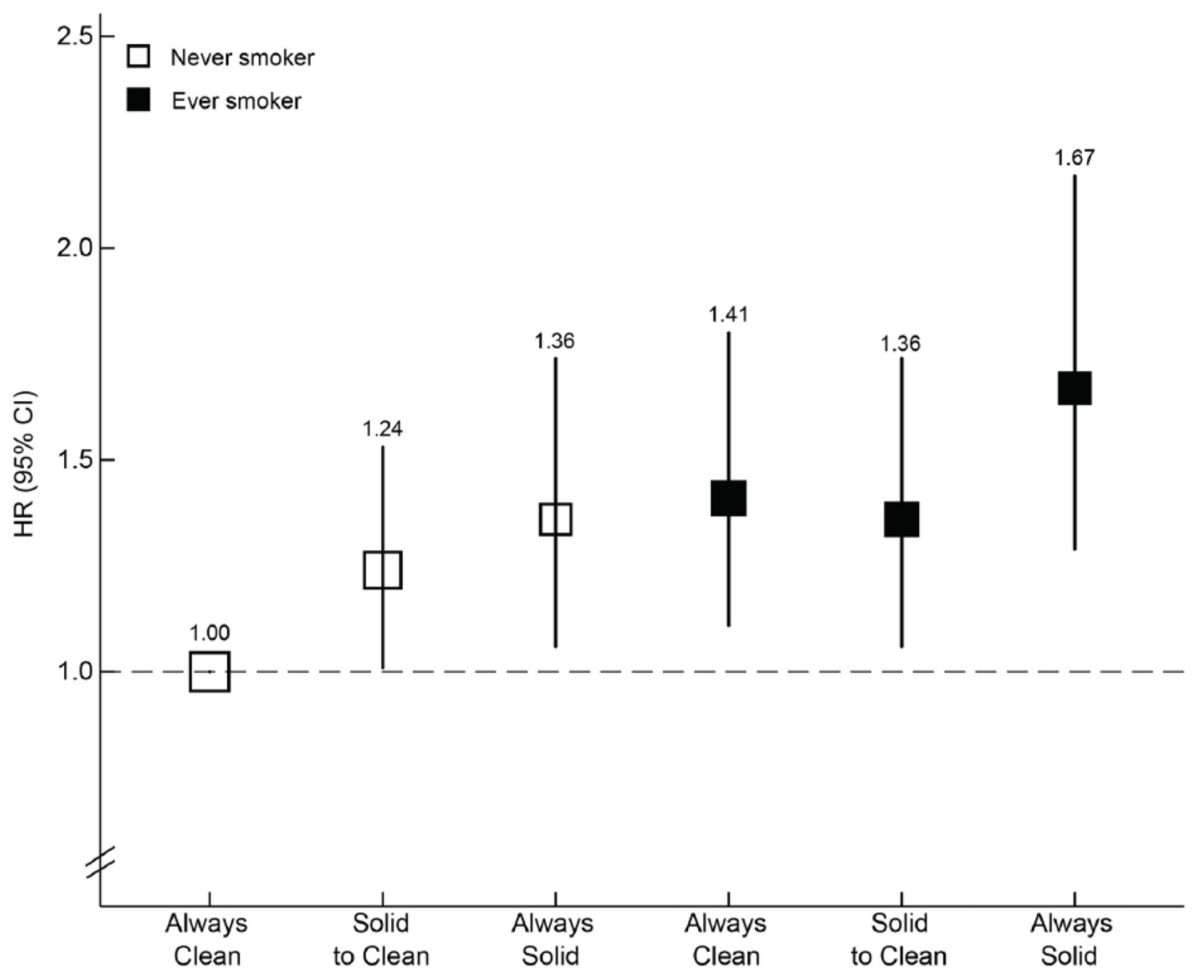
Associations of smoking status and long-term cooking fuel exposure with liver cancer risk. HRs were stratified by sex, baseline age groups, study regions and adjusted for education, household income, occupation, marital status, alcohol consumption, environmental tobacco smoke, months of storing pesticides at home, long-term heating fuel exposure, stoves with chimney/extractor, physical activity, BMI, having a refrigerator at home, consumption frequency of fresh fruit, preserved vegetables, red meat, fish and grains at baseline, hepatitis B test result, family history of cancer, medical history of hepatic cirrhosis and diabetes. The size of the box was inversely proportional to the variance of the logarithm of the category-specific log risk, and the vertical lines represent 95% CIs. The numbers above the vertical lines were point estimates for HRs. The analysis was restricted to individuals who had data on solid fuel use and smoking (N=373,448).

As shown in Figure 1, compared with never smokers who had always used clean fuels, ever smokers who had always used solid fuels had the highest risk of liver cancer (HRs: 1.67, 95% CIs: 1.31-2.19). Both ever smokers who had used clean fuels and never smokers who had used solid fuels had smaller but significantly elevated risks, with HRs of 1.41 (1.11-1.80) and 1.36 (1.09-1.78), respectively.

In the subgroup analyses, the association with current smoking appeared greater in HBsAg seropositive participants (HRs: 1.35, 95% CIs: 1.09-1.68 vs. 1.26, 1.11-1.43; P_interaction_= 0.042) and ever-regular drinkers (1.42, 1.25-1.62 vs. 1.13, 0.94-1.37; P_interaction_=0.031) than their counterparts (Table 3). No significant interaction was found between the amount of daily cigarette consumption and liver cancer across all stratums (Supplementary Table 1). When stratified by sex, solid fuel use remained significantly and positively associated with liver cancer risk in females (1.59, 1.14-2.20), but not in males (1.13, 0.89-1.45; P_interaction_<0.001). The positive relationship between solid fuel types and liver cancer risks was much more pronounced among rural participants, with 51% (1.05-2.19), 57% (1.11-2.22), and 70% (1.17-2.46) elevated risks for coal, wood, and mix solid fuel users, respectively, but not among urban citizens (P_interaction_=0.011, Supplementary Table 2).

The associations of smoking and solid fuel use with liver cancer risks remained consistent with the main results after adjusting for 11 groups of alcohol consumption, excluding liver cancer cases within the first 2 years of follow-up and those who had family history of cancer (Supplementary Table 3).

## Discussion

In this large prospective study, we found that tobacco smoking and long-term solid fuel use for cooking were both associated with higher risk of liver cancer, and these associations were stronger with higher daily cigarette consumption, earlier starting age of regular smoking, and total duration of solid fuels exposure. HBV infection, alcohol drinking, sex and area of residence are potential effect modifiers.

The present study corroborates previous findings on smoking and liver cancer [7, 24]. A meta-analysis including 24 cohort studies published till 2016 found that regular smokers had 66% (95% CIs: 1.53-1.80) higher risk of hepatocellular carcinoma (HCC) [7]. Another meta-analysis found similar results, with a pooled OR of 1.51 (95% CIs: 1.37-1.67) for liver cancer associated with current smoking [24]. Both reviews reported higher risk of liver cancer associated with smoking than observed that discovered in the present study. Such difference could partly be explained by the study population. The studies enrolled in the meta-analyses were mainly conducted in developed countries, where the smoking epidemic had reached its summit. We have further examined the dose-response associations with the number of cigarettes consumed on the risk of liver cancer. Previous cohort studies conducted in China found similar results but with larger magnitude, with RRs of smoking > 20 cigarettes per day ranging from 1.60 to1.80 [14, 25]. However, one only enrolled males [14] while the other investigated liver cancer mortality in city factory workers [25].

For solid fuels combustion, although previous reports mainly focused on its association with cardiorespiratory diseases [26], a recent study suggested that the liver might also be affected by air pollutants due to its role in detoxification [27]. A case-control study including 314 HCC cases and 368 controls in China reported a 3.91-fold HCC risk (95% CIs: 2.62-5.83) among those exposed to indoor air pollution [28]. However, it lacked a detailed definition of indoor air pollution and only adjusted for education. In the present study, we observed 25% higher risk of liver cancer incidence among those who had always used solid fuels for cooking, and further identified apparently greater hazard in persistent wood users than coal users. A possible explanation might be that wood combustion produced much higher levels of air pollutants [especially particulate matter (PM), many of which are carcinogens such as polycyclic aromatic hydrocarbons (PAHs)] than coal [29].

The associations between tobacco smoking and liver cancer stratified by HBV infection status have been controversial. Similar to this study, a meta-analysis of 9 studies (5 case-control and 4 cohort studies) [30] and a recent study of 2,011 liver cancer cases and 7,933 controls in China [31] found that the association of smoking with liver cancer appeared stronger in HBV positive individuals. In contrast, some studies observed significant association only among HBV negative participants [32–34], but all of them were case-control studies with less than 200 cases. When stratified by alcohol drinking status, smoking remained significantly and positively associated with liver cancer risk among ever drinkers, but not among never drinkers. Previously two hospital-based case-control studies conducted in Greece and United States had found similar results, with 6-9-fold higher risk of HCC among those exposed to both drinking and smoking compared with those exposed to neither [35, 36]. The interaction is biologically plausible since the IARC had identified both tobacco and alcoholic beverages as group 1 human carcinogens for liver cancer [37, 38].

The association of solid fuel use with liver cancer incidence appeared greater in females than in males, possibly because females tend to cook much more regularly and intensely in China [39]. The positive association between solid fuel types and liver cancer risks remained significant among rural but not urban residents. This could be due to poorer ventilation and lower quality fuels and thus higher exposure to noxious pollutants in rural residents [40].

Solid fuels combustion could produce a large amount of air pollutants, especially PM and PAHs, which have been reported to induce the development and progression of liver cancer [41]. Our previous work found long-term solid fuel use for cooking and smoking to be independently associated with a higher risk of death from chronic liver diseases [16]. Similarly, the positive relationship between solid fuels for cooking and incident liver cancer risk was much more pronounced among ever smokers than never smokers.

Our finding highlighted the double burden from tobacco smoking and solid fuels on liver cancer in China. These two risk factors are both more prevalent in less-developed areas, and greater attention should be placed on smoking cessation and promotion of access to clean energy to reduce the burden caused by liver cancer, among other diseases [42, 43]. Notably, no significant excess risk of liver cancer incidence was found among those who had switch from solid to clean fuels compared with long-term clean fuel users, supporting the potential benefits of clean fuel transition. In addition, chronic carriers of HBV and alcohol drinkers, who are also more likely to smoke [44], should be suggested to avoid smoking.

To the best of our knowledge, the present study was among the first prospective studies to investigate the independent and joint impact of smoking and solid fuel use for cooking on liver cancer incidence among Chinese adults. The strengths of this study included the large sample size, prospective cohort design, and long-term follow-up via robust record linkage to hospitalization records. The comprehensive survey in CKB collected a range of risk factors for liver cancer, including hepatitis B infection status and history of hepatic cirrhosis, enabling adjustment for important confounders in our analyses. However, some limitations merit discussion. First, smoking habits and solid fuel use were self-reported, which might entail recall and/or social desirability bias. Second, although we used a comprehensive questionnaire to measure both exposures, we lacked more detailed information for cooking fuel use, such as stove types, secondary fuel type used, and intra-day cooking frequency, which prevented us from getting more precise exposure classification. Although these data were unavailable, we adjusted for stove ventilation condition and excluded those who cooked irregularly at baseline to reduce noise and confounding. Third, we had no data on aflatoxins exposure, which is a strong risk factor for liver cancer [45]. Aflatoxins are usually produced from improper storage of food (corns, peanuts, and spices, etc.) in warm and humid regions, and it is typically associated with lower socioeconomic status [46]. All analyses in this study were stratified by the 10 study areas and adjusted for a range of socioeconomic factors in order to reduce the confounding. Fourth, incident liver cancer cases were documented via linkage to death and disease registries and hospitalization records. Under-diagnosis might exist due to poor healthcare access or health awareness associated with lower socioeconomic status and/or rural residency, both of which were positively associated with higher solid fuel use in CKB, so the associations observed may be underestimated.

## Conclusion

Tobacco smoking and solid fuel use for cooking were each independently associated with higher risk of liver cancer incidence. The strength of the association increased with the amount of daily cigarette consumption, earlier age of starting smoking, and total duration of solid fuels exposure. Our findings reinforce the importance of smoking cessation and promotion of access to clean energy, especially in less-developed areas.

## Supplementary Material

Supplementary Appendix

## Figures and Tables

**Table 1:** Baseline characteristics of participants according to smoking status and long-term cooking fuel use.

	Smoking status			Cooking fuel		
	Never	Ex-smoker	Current smoker	Always clean	Solid to clean	Always solid
N	343,198	15,175	149,464	86,782	104,381	182,285
Age (years)	51.5	56.6	52.8	46.0	52.4	54.6
Female (%)	84.6	8.0	5.5	59.5	80.5	77.5
Rural (%)	52.7	50.7	63.2	12.2	18.2	91.1
Married (%)	91.1	91.8	88.7	88.5	89.7	89.1
Middle school and above (%)	51.3	48.2	44.7	62.7	52.6	38.3
Household income > 20000 yuan/year (%)	44.1	42.8	39.9	55.3	47.3	30.2
Occupation (%)						
Manual	54.7	53.3	58.5	39.5	39.0	68.4
Non-manual	14.0	12.9	11.1	17.6	14.9	5.8
Not working	31.3	33.8	30.4	42.9	46.0	25.8
Passive smoker (%)	42.6	44.4	48.0	44.8	48.1	51.3
Current alcohol drinker (%)	9.5	17.1	18.7	10.9	10.9	11.2
Current smoker (%)	-	-	-	17.9	18.9	20.1
Ventilation with all stoves (%)	45.3	43.8	42.6	65.3	60.3	26.8
Heating fuel type (%)						
Always clean	8.9	8.5	8.0	18.5	7.1	4.5
Always solid	35.7	36.1	36.8	21.7	24.8	43.2
Not heating	43.0	42.2	42.2	43.3	43.6	45.7
Storing pesticide (months)	4.1	4.1	4.2	2.3	2.7	5.2
Physical activity (MET-h/d)	21.0	20.9	21.4	18.6	19.7	22.5
Overweight and obesity (%)	45.9	51.2	38.2	47.6	51.4	40.6
HBsAg Positive (%)	3.0	2.8	3.1	2.7	2.9	3.1
History of diabetes (%)	5.9	6.3	5.8	6.8	7.2	4.9
History of hepatic cirrhosis (%)	1.3	1.1	1.2	1.1	1.3	0.9

Values are means or percentages of participants adjusted for age, sex and region, where appropriate. Abbreviations: HBsAg, hepatitis B surface antigen; MET-h/d, metabolic equivalent of tasks-hours per day

**Table 2:** Hazard ratios for liver cancer by smoking characteristics and long-term cooking fuel use.

	N (Rates /100,000 PYs)	HRs (95% CIs) a		
		Model 1: basic adjustment	Model 2: basic + lifestyle factors	Model 3: basic + lifestyle + medical factors
**Smoking status**				
Never **(common reference)**	1,374 (36.6)	1.00	1.00	1.00
Ex-smoker	153 (95.8)	1.15 (0.96,1.39)	1.12 (0.93,1.35)	1.13 (0.94,1.36)
Current smoker	1,470 (92.8)	1.33 (1.20,1.48)	1.26 (1.13,1.40)	1.28 (1.15,1.42)
**No. of cigarettes/day**				
≤10	532 (91.4)	1.24 (1.10,1.40)	1.19 (1.05,1.35)	1.19 (1.05,1.35)
11-20	761 (91.8)	1.35 (1.20,1.52)	1.28 (1.14,1.44)	1.30 (1.15,1.47)
>20	330 (99.4)	1.38 (1.19,1.59)	1.27 (1.10,1.48)	1.32 (1.14,1.54)
P_trend_		<0.001	<0.001	<0.001
**Age started smoking (years)**				
>25	372 (98.0)	1.20 (1.05,1.37)	1.16 (1.01,1.33)	1.18 (1.03,1.36)
19-25	775 (90.2)	1.31 (1.17,1.47)	1.24 (1.10,1.40)	1.26 (1.12,1.42)
≤18	476 (94.3)	1.43 (1.25,1.62)	1.33 (1.16,1.52)	1.34 (1.17,1.53)
P_trend_		<0.001	0.003	0.001
**Long-term fuel type**				
Always clean **(common reference)**	363 (38.5)	1.00	1.00	1.00
Solid to clean	513 (45.2)	1.09 (0.94,1.27)	1.12 (0.96,1.30)	1.10 (0.94,1.28)
Always solid	1,009 (50.7)	1.29 (1.07,1.55)	1.23 (1.01,1.49)	1.25 (1.03,1.52)
**Types of solid fuels b**				
Always coal	356 (45.2)	1.17 (0.91,1.50)	1.15 (0.89,1.48)	1.14 (0.88,1.47)
Always wood	480 (56.7)	1.31 (1.08,1.58)	1.24 (1.01,1.51)	1.27 (1.04,1.56)
Always solid (mixed)	173 (48.7)	1.39 (1.08,1.79)	1.33 (1.03,1.72)	1.34 (1.04,1.73)
**Duration exposed (years)**				
1-14	322 (42.6)	1.05 (0.89,1.24)	1.06 (0.90,1.26)	1.06 (0.89,1.25)
15-29	498 (41.6)	1.17 (1.00,1.37)	1.18 (1.00,1.38)	1.16 (0.99,1.37)
≥30	702 (60.0)	1.23 (1.04,1.45)	1.22 (1.03,1.44)	1.21 (1.03,1.44)
P_trend_		0.008	0.018	0.018

a HRs were stratified by sex, baseline age groups and study regions, model 1 were adjusted for education, household income, occupation and marital status; model 2 were additionally adjusted for alcohol consumption, environmental tobacco smoke, months of storing pesticides at home, long-term heating fuel exposure, stoves with chimney/extractor, physical activity, BMI, having a refrigerator at home, consumption frequency of fresh fruit, preserved vegetables, red meat, fish and grains at baseline, and mutually adjusted for long-term cooking fuel exposure and smoking habits; model 3 were additionally adjusted for hepatitis B test result, family history cancer, medical history of hepatic cirrhosis and diabetes.

b Analysis was restricted to individuals who always solid fuels in the last 3 residences before the baseline survey (N=182,285).

**Table 3:** Hazard ratios for liver cancer associated with smoking status and long-term cooking fuel use, stratified by baseline characteristics.

	Smoking status			P_interact_	Cooking fuel type			P_interact_
	Never	Ex-smoker	Current smoker		Always clean	Solid to clean	Always solid	
Sex				0.803				<0.001
Male	1.00	1.16 (0.95,1.40)	1.30 (1.15,1.46)		1.00	1.01 (0.83,1.22)	1.13 (0.89,1.45)	
Female	1.00	0.82 (0.36,1.86)	1.20 (0.90,1.60)		1.00	1.34 (1.03,1.75)	1.59 (1.14,2.20)	
Region				0.788				0.092
Rural	1.00	1.29 (0.99,1.69)	1.37 (1.18,1.58)		1.00	1.28 (0.86,1.90)	1.60 (1.12,2.20)	
Urban	1.00	1.07 (0.83,1.37)	1.23 (1.05,1.44)		1.00	1.01 (0.87,1.19)	1.08 (0.82,1.41)	
HBsAg				0.042				0.065
Negative	1.00	1.06 (0.85,1.33)	1.26 (1.11,1.43)		1.00	1.11 (0.92,1.34)	1.41 (1.12,1.78)	
Positive	1.00	1.33 (0.91,1.93)	1.35 (1.09,1.68)		1.00	1.08 (0.81,1.46)	0.84 (0.56,1.27)	
Cirrhosis				0.621				0.833
No	1.00	1.16 (0.95,1.41)	1.29 (1.15,1.45)		1.00	1.10 (0.94,1.29)	1.24 (1.01,1.52)	
Yes	1.00	1.00 (0.56,1.77)	1.20 (0.87,1.66)		1.00	1.17 (0.70,1.96)	1.71 (0.88,3.30)	
Drinker				0.031				0.209
Never	1.00	1.02 (0.70,1.49)	1.13 (0.94,1.37)		1.00	1.31 (1.02,1.69)	1.45 (1.08,1.96)	
Ever	1.00	1.25 (1.01,1.54)	1.42 (1.25,1.62)		1.00	0.97 (0.80,1.18)	1.12 (0.86,1.46)	
BMI (kg/m^2^)				0.546				0.117
18.5-24.0	1.00	1.12 (0.85,1.48)	1.37 (1.18,1.59)		1.00	1.30 (1.03,1.64)	1.48 (1.13,1.94)	
≥24	1.00	1.07 (0.83,1.39)	1.19 (1.01,1.40)		1.00	0.97 (0.79,1.19)	0.97 (0.71,1.33)	
Physical activity				0.876				0.516
Low	1.00	1.15 (0.90,1.47)	1.35 (1.17,1.57)		1.00	1.12 (0.92,1.37)	1.38 (1.05,1.81)	
High	1.00	1.11 (0.84,1.46)	1.21 (1.03,1.41)		1.00	1.04 (0.82,1.32)	1.11 (0.84,1.47)	

HRs were stratified by sex, baseline age groups, study regions and adjusted for education, household income, occupation, marital status, alcohol consumption, environmental tobacco smoke, months of storing pesticides at home, long-term heating fuel exposure, stoves with chimney/extractor, physical activity, BMI, having a refrigerator at home, consumption frequency of fresh fruit, preserved vegetables, red meat, fish and grains at baseline, hepatitis B test result, family history of cancer, medical history of hepatic cirrhosis and diabetes, mutually adjusted for long-term cooking fuel exposure and smoking habits. Abbreviations: BMI, Body mass index; HBsAg, hepatitis B surface antigen

## Data Availability

The dataset for this study is available at www.ckbiobank.org, as well as the access policy and procedures. Further information is available from the corresponding author upon request.

## Abbreviations

BMI: Body mass index
CKB: China Kadoorie Biobank
HBsAg: hepatitis B surface antigen
HBV: hepatitis B virus
HCC: hepatocellular carcinoma
HCV: hepatitis C virus
IARC: International Agency for Research on Cancer
MET-h/d: metabolic equivalent hours per day
