# Supplementary material for: Tobacco smoking and solid fuels for cooking and risk of liver cancer: A prospective cohort study of 0.5 million Chinese adults
Source: Int J Cancer. 2022 Mar 3;151(2):181–90. doi: 10.1002/ijc.33977 (PMC7612779; doi:10.1002/ijc.33977)
Supplement: Supplementary file 1 — Appendix S1 Supporting Information. [file IJC-151-181-s001.pdf]

Tobacco smoking and solid fuels for cooking and risk of liver cancer: a prospective cohort study of 0.5 million Chinese adults

Qiaorui Wen, Ka Hung Chan, Kexiang Shi, Jun Lv, Yu Guo, Pei Pei, Ling Yang, Yiping Chen, Huaidong Du, Simon Gilbert, Daniel Avery, Weijie Hu, Junshi Chen, Canqing Yu, Zhengming Chen, Liming Li

Members of the China Kadoorie Biobank collaborative group……………………………………………………………………………………………….2
Supplementary table 1. HRs for liver cancer associated with smoking characteristics, stratified by baseline characteristics..…………………………………………….3
Supplementary table 2. HRs for liver cancer associated with long-term cooking fuel use, stratified by baseline characteristics………………………………………..…4
Supplementary table 3. Sensitivity analysis for adjusted hazard ratios for liver cancer associated with smoking characteristics and long-term cooking fuel use…….5

Members of the China Kadoorie Biobank collaborative group

International Steering Committee: Junshi Chen, Zhengming Chen (PI), Robert Clarke, Rory Collins, Yu Guo, Liming Li (PI), Jun Lv, Richard Peto, Robin Walters. **International Co-ordinating Centre, Oxford:** Daniel Avery, Ruth Boxall, Derrick Bennett, Yumei Chang, Yiping Chen, Zhengming Chen, Robert Clarke, Huaidong Du, Simon Gilbert, Alex Hacker, Mike Hill, Michael Holmes, Andri Iona, Christiana Kartsonaki, Rene Kerosi, Ling Kong, Om Kurmi, Garry Lancaster, Sarah Lewington, Kuang Lin, John McDonnell, Iona Millwood, Qunhua Nie, Jayakrishnan Radhakrishnan, Paul Ryder, Sam Sansome, Dan Schmidt, Paul Sherliker, Rajani Sohoni, Becky Stevens, Iain Turnbull, Robin Walters, Jenny Wang, Lin Wang, Neil Wright, Ling Yang, Xiaoming Yang. **National Co-ordinating Centre, Beijing:** Yu Guo, Xiao Han, Can Hou, Jun Lv, Pei Pei, Chao Liu, Canqing Yu. **10 Regional Co-ordinating Centres: Qingdao CDC:** Zengchang Pang, Ruqin Gao, Shanpeng Li, Shaojie Wang, Yongmei Liu, Ranran Du, Yajing Zang, Liang Cheng, Xiaocao Tian, Hua Zhang, Yaoming Zhai, Feng Ning, Xiaohui Sun, Feifei Li. **Licang CDC:** Silu Lv, Junzheng Wang, Wei Hou. **Heilongjiang Provincial CDC:** Mingyuan Zeng, Ge Jiang, Xue Zhou. **Nangang CDC:** Liqiu Yang, Hui He, Bo Yu, Yanjie Li, Qinai Xu,Quan Kang, Ziyan Guo. **Hainan Provincial CDC:** Dan Wang, Ximin Hu, Jinyan Chen, Yan Fu, Zhenwang Fu, Xiaohuan Wang. **Meilan CDC:** Min Weng, Zhendong Guo, Shukuan Wu,Yilei Li, Huimei Li, Zhifang Fu. **Jiangsu Provincial CDC:** Ming Wu, Yonglin Zhou, Jinyi Zhou, Ran Tao, Jie Yang, Jian Su. **Suzhou CDC:** Fang liu, Jun Zhang, Yihe Hu, Yan Lu, , Liangcai Ma, Aiyu Tang, Shuo Zhang, Jianrong Jin, Jingchao Liu. **Guangxi Provincial CDC:** Zhenzhu Tang, Naying Chen, Ying Huang. **Liuzhou CDC:** Mingqiang Li, Jinhuai Meng, Rong Pan, Qilian Jiang, Jian Lan,Yun Liu, Liuping Wei, Liyuan Zhou, Ningyu Chen Ping Wang, Fanwen Meng, Yulu Qin,, Sisi Wang. **Sichuan Provincial CDC:** Xianping Wu, Ningmei Zhang, Xiaofang Chen,Weiwei Zhou. **Pengzhou CDC:** Guojin Luo, Jianguo Li, Xiaofang Chen, Xunfu Zhong, Jiaqiu Liu, Qiang Sun. **Gansu Provincial CDC:** Pengfei Ge, Xiaolan Ren, Caixia Dong. **Maiji CDC:** Hui Zhang, Enke Mao, Xiaoping Wang, Tao Wang, Xi zhang. **Henan Provincial CDC:** Ding Zhang, Gang Zhou, Shixian Feng, Liang Chang, Lei Fan. **Huixian CDC:** Yulian Gao, Tianyou He, Huarong Sun, Pan He, Chen Hu, Xukui Zhang, Huifang Wu, Pan He. **Zhejiang Provincial CDC:** Min Yu, Ruying Hu, Hao Wang. Tongxiang CDC: Yijian Qian, Chunmei Wang, Kaixu Xie, Lingli Chen, Yidan Zhang, Dongxia Pan, Qijun Gu. **Hunan Provincial CDC:** Yuelong Huang, Biyun Chen, Li Yin, Huilin Liu, Zhongxi Fu, Qiaohua Xu. **Liuyang CDC:** Xin Xu, Hao Zhang, Huajun Long, Xianzhi Li, Libo Zhang, Zhe Qiu.

Supplementary table 1. HRs for liver cancer associated with smoking characteristics, stratified by baseline characteristics.

	No. of cigarettes/day			$P_{interact}$	Age started smoking (years)			$P_{interact}$
	≤10	11-20	>20		>25	19-25	≤18	
Gender				0.826				0.948
Male	1.21 (1.06,1.39)	1.31 (1.15,1.49)	1.33 (1.14,1.55)		1.21 (1.04,1.40)	1.27 (1.12,1.45)	1.35 (1.18,1.56)	
Female	1.08 (0.78,1.49)	1.25 (0.74,2.08)	2.05 (0.76,5.54)		1.03 (0.68,1.54)	1.16 (0.72,1.89)	1.34 (0.86,2.09)	
Region				0.742				0.152
Rural	1.27 (1.08,1.50)	1.45 (1.23,1.71)	1.41 (1.15,1.71)		1.18 (0.98,1.43)	1.41 (1.20,1.65)	1.48 (1.24,1.76)	
Urban	1.18 (0.98,1.43)	1.19 (1.00,1.42)	1.27 (1.01,1.60)		1.23 (1.01,1.50)	1.13 (0.95,1.35)	1.30 (1.06,1.59)	
HBsAg				0.083				0.368
Negative	1.14 (0.98,1.32)	1.28 (1.10,1.47)	1.41 (1.19,1.69)		1.16 (0.99,1.37)	1.24 (1.08,1.43)	1.32 (1.12,1.55)	
Positive	1.35 (1.06,1.73)	1.40 (1.11,1.78)	1.19 (0.87,1.63)		1.19 (0.89,1.59)	1.36 (1.08,1.73)	1.47 (1.14,1.91)	
Cirrhosis				0.355				0.688
No	1.22 (1.07,1.39)	1.30 (1.14,1.48)	1.36 (1.16,1.60)		1.20 (1.04,1.39)	1.28 (1.13,1.46)	1.35 (1.17,1.55)	
Yes	0.96 (0.65,1.42)	1.44 (1.01,2.05)	1.02 (0.63,1.66)		1.07 (0.67,1.71)	1.12 (0.79,1.61)	1.34 (0.90,1.98)	
Drinker				0.086				0.046
Never	1.04 (0.83,1.31)	1.18 (0.95,1.47)	1.21 (0.89,1.65)		1.04 (0.80,1.34)	1.19 (0.96,1.47)	1.11 (0.86,1.44)	
Ever	1.31 (1.13,1.53)	1.44 (1.25,1.66)	1.50 (1.26,1.78)		1.30 (1.10,1.53)	1.38 (1.20,1.59)	1.55 (1.32,1.81)	
BMI (kg/m^2)				0.778				0.435
18.5-24.0	1.27 (1.07,1.51)	1.38 (1.17,1.63)	1.45 (1.19,1.78)		1.26 (1.04,1.52)	1.32 (1.11,1.55)	1.49 (1.25,1.78)	
≥24	1.12 (0.92,1.36)	1.19 (0.99,1.44)	1.23 (0.97,1.56)		1.14 (0.92,1.41)	1.18 (0.98,1.41)	1.20 (0.97,1.49)	
Physical activity				0.373				0.776
Low	1.28 (1.08,1.52)	1.38 (1.17,1.62)	1.28 (1.04,1.58)		1.30 (1.08,1.57)	1.30 (1.10,1.53)	1.39 (1.16,1.67)	
High	1.10 (0.92,1.32)	1.23 (1.03,1.46)	1.38 (1.11,1.71)		1.08 (0.88,1.32)	1.22 (1.02,1.45)	1.29 (1.06,1.56)	

Taking those who had never smoked regularly as common reference group.

HRs were stratified by gender, baseline age groups and study regions and adjusted for education, household income, occupation, marital status, alcohol consumption, environmental tobacco smoke, months of storing pesticides at home, long-term cooking and heating fuel exposure, stoves with chimney/extractor, physical activity, BMI, having a refrigerator at home, consumption frequency of fresh fruit, preserved vegetables, red meat, fish and grains at baseline, hepatitis B test result, family history of cancer, medical history of hepatic cirrhosis and diabetes.

Abbreviations: HBsAg, hepatitis B surface antigen; BMI, Body mass index

Supplementary table 2. HRs for liver cancer associated with long-term cooking fuel use, stratified by baseline characteristics.

	Cooking fuel type (Always solid) [a]			$P_{interact}$	Duration exposed (years)			$P_{interact}$
	Always coal	Always wood	Solid (mixed)		1-14	15-29	≥30	
Gender				<0.001				0.007
Male	1.02 (0.73,1.43)	1.12 (0.86,1.45)	1.84 (1.25,2.71)		0.95 (0.77,1.17)	0.99 (0.80,1.22)	1.25 (1.01,1.55)	
Female	1.52 (1.01,2.28)	1.63 (1.16,2.28)	1.45 (0.99,2.12)		1.33 (0.99,1.78)	1.48 (1.12,1.95)	1.32 (0.99,1.76)	
Region				0.011				0.055
Rural	1.51 (1.05,2.19)	1.57 (1.11,2.22)	1.70 (1.17,2.46)		1.32 (0.92,1.90)	1.57 (1.11,2.22)	1.60 (1.13,2.28)	
Urban	0.43 (0.19,0.97)	1.28 (0.95,1.71)	0.85 (0.35,2.07)		0.98 (0.80,1.18)	1.01 (0.84,1.23)	1.11 (0.90,1.37)	
HBsAg				0.183				0.043
Negative	1.30 (0.96,1.74)	1.44 (1.14,1.83)	1.45 (1.08,1.95)		1.12 (0.91,1.37)	1.19 (0.98,1.45)	1.26 (1.03,1.53)	
Positive	0.72 (0.40,1.31)	0.86 (0.56,1.33)	1.04 (0.55,1.94)		0.96 (0.69,1.34)	1.02 (0.73,1.42)	1.16 (0.82,1.64)	
Cirrhosis				0.776				0.974
No	1.18 (0.90,1.54)	1.25 (1.01,1.54)	1.27 (0.97,1.66)		1.06 (0.89,1.27)	1.16 (0.97,1.38)	1.20 (1.01,1.44)	
Yes	0.66 (0.24,1.78)	1.96 (0.98,3.90)	3.72 (1.43,9.66)		1.03 (0.58,1.83)	1.46 (0.86,2.51)	1.51 (0.84,2.73)	
Drinker				0.324				0.253
Never	1.21 (0.82,1.80)	1.50 (1.11,2.04)	1.43 (0.98,2.07)		1.28 (0.97,1.69)	1.40 (1.08,1.83)	1.36 (1.04,1.78)	
Ever	1.09 (0.77,1.53)	1.11 (0.84,1.47)	1.28 (0.89,1.85)		0.93 (0.75,1.15)	1.00 (0.81,1.24)	1.14 (0.91,1.42)	
BMI (kg/m^2)				0.200				0.332
18.5-24.0	1.30 (0.92,1.84)	1.53 (1.16,2.02)	1.46 (1.03,2.07)		1.27 (0.99,1.62)	1.36 (1.07,1.74)	1.46 (1.14,1.87)	
≥24	0.98 (0.64,1.49)	0.94 (0.67,1.32)	1.12 (0.74,1.72)		0.90 (0.71,1.15)	1.02 (0.81,1.29)	1.00 (0.78,1.29)	
Physical activity				0.076				0.602
Low	1.32 (0.93,1.86)	1.33 (1.00,1.78)	1.85 (1.30,2.64)		1.04 (0.83,1.31)	1.20 (0.96,1.49)	1.35 (1.07,1.69)	
High	1.01 (0.69,1.48)	1.13 (0.85,1.49)	0.94 (0.65,1.39)		1.05 (0.81,1.35)	1.10 (0.85,1.41)	1.04 (0.80,1.34)	

Taking those always used clean fuels for cooking as common reference group.

[a] Analysis was restricted to individuals who always solid fuels in the last 3 residences before baseline survey (N=182,285).

HRs were stratified by gender, baseline age groups and study regions and adjusted for education, household income, occupation, marital status, alcohol consumption, smoking habits, environmental tobacco smoke, months of storing pesticides at home, long-term heating fuel exposure, stoves with chimney/extractor, physical activity, BMI, having a refrigerator at home, consumption frequency of fresh fruit, preserved vegetables, red meat, fish and grains at baseline, hepatitis B test result, family history of cancer, medical history of hepatic cirrhosis and diabetes.

Abbreviations: HBsAg, hepatitis B surface antigen; BMI, Body mass index

Supplementary table 3. Sensitivity analysis for adjusted hazard ratios for liver cancer associated with smoking characteristics and long-term cooking fuel use.

		Smoking status			Cooking fuel		
		Never	Ex-smoker	Current smoker	Always clean	Solid to clean	Always solid
Main analysis							
	No. of cases	1,374	153	1,470	365	513	1,009
	HR (95% CIs)	1.00	1.13 (0.94,1.36)	1.28 (1.15,1.42)	1.00	1.10 (0.94,1.28)	1.25 (1.03,1.52)
Excluding first 2 years of follow-up							
	No. of cases	1,189	130	1,231	309	441	848
	HR (95% CIs)	1.00	1.15 (0.94,1.40)	1.28 (1.14,1.44)	1.00	1.11 (0.94,1.31)	1.26 (1.02,1.55)
Excluding family history of cancer							
	No. of cases	1,125	120	1,174	279	401	848
	HR (95% CIs)	1.00	1.14 (0.93,1.41)	1.30 (1.15,1.46)	1.00	1.14 (0.96,1.36)	1.34 (1.08,1.66)
Adjustment for 11 groups of alcohol consumption							
	No. of cases	1,374	153	1,470	365	513	1,009
	HR (95% CIs)	1.00	1.14 (0.95,1.37)	1.28 (1.15,1.43)	1.00	1.09 (0.94,1.27)	1.25 (1.03,1.51)

HRs were stratified by gender, baseline age groups and study regions and adjusted for education, household income, occupation, marital status, alcohol consumption, environmental tobacco smoke, months of storing pesticides at home, long-term heating fuel exposure, stoves with chimney/extractor, physical activity, BMI, having a refrigerator at home, consumption frequency of fresh fruit, preserved vegetables, red meat, fish and grains at baseline, hepatitis B test result, family history of cancer, medical history of hepatic cirrhosis and diabetes, mutually adjusted for long-term cooking fuel exposure and smoking habits.
